# Is assessment of oral health-related quality of life burdensome? An item nonresponse analysis of the oral health impact profile

**DOI:** 10.1186/s12903-021-01954-w

**Published:** 2021-11-23

**Authors:** Swaha Pattanaik, Chi Hyun Lee, Mike T. John, Phonsuda Chanthavisouk, Danna Paulson

**Affiliations:** 1 Department of Diagnostic and Biological Sciences, School of Dentistry, University of Minnesota, 515 Delaware Street Southeast, Minneapolis, MN 55455-0348 USA; 2 Department of Biostatistics and Epidemiology, School of Public Health & Health Sciences, University of Massachusetts, Amherst, MA USA; 3 Department of Primary Dental Care, School of Dentistry, University of Minnesota, Minneapolis, MN USA

**Keywords:** Missing data, Oral health-related quality of life, Oral health impact profile, Patient-reported outcome measures

## Abstract

**Aim:**

This study aimed to investigate if in the 49-item Oral Health Impact Profile (OHIP): (i) more missing data occurred when participants answered more questions, (ii) more missing data occurred in a particular item or set of related items, and (iii) item missingness was associated with the demographic characteristics and oral health-related quality of life (OHRQoL) impairment level.

**Methods:**

We used OHIP data from the Dimensions of OHRQoL (DOQ) project, which consolidated data from 35 individual studies. Among these studies, we analyzed OHIP data from 19 studies (4,847 surveyed individuals, of which 3,481 were completed under supervision and 1,366 were completed unsupervised) that contained some missing information. We computed descriptive statistics to investigate the OHIP missingness. We also used logistic regression analyses, with missing information as the dependent variable, and number of questions filled in (OHIP item rank) as the independent variable for samples with and without supervision. To investigate whether missing data occurs more in a particular item or set of related items we fitted regression models with individual OHIP items and the OHRQoL dimensions as indicator variables. We also investigated age, gender, and OHRQoL level as predictor variables for missing OHIP items.

**Results:**

We found very low levels of missingness across individual OHIP items and set of related items, and there was no particular item or set of related items that was associated with more missing data. Also, more missing data did not depend on whether the participants answered more questions. In studies without supervision, older persons and females were 5.47 and 2.66 times more likely to have missing items than younger persons and females. However, in studies with supervision, older persons, and participants with more OHRQoL impairment were 1.70 and 2.65 times more likely to have missing items.

**Conclusion:**

The study participants from general and dental patient populations did not find OHIP-49 burdensome. OHIP item missingness did not depend on a particular OHIP item or set of related items, or if the study participants responded to a greater number of OHIP items. We did not find a consistent pattern of the influence of sociodemographic and OHRQoL magnitude information on OHIP missingness. The amount of missing OHIP information was low making any potential influence likely small in magnitude.

## Introduction

Oral health-related quality of life (OHRQoL) is a well-recognized concept that represents how patients perceive the impact of oral diseases and dental interventions [1–3]. Oral health professionals measure OHRQoL to assess the burden of oral diseases on patients, and to help identify the dental treatments which are the most effective in reducing the burden [3]. OHRQoL is one of the many dental patient-reported outcomes (dPROs) used to assess the impact of oral diseases [4–6]. Several instruments or dental patient-reported outcome measures (dPROMs) such as the 49-item Oral Health Impact Profile (OHIP-49), the General Oral Health Assessment Index (GOHAI), and the Oral Impacts on Daily Performances (OIDP) are available to measure OHRQoL [4, 6].

When dentists and researchers administer lengthy OHRQoL instruments to patients or research participants, the information is often incomplete and/or missing. They find missing information for either all the items (subject nonresponse), or for specific items (item nonresponse). Consequently, subject and item nonresponse challenges the validity of their study findings. Subject nonresponse is more challenging to identify as typically it is not known which person chose not to participate in the research. In contrast, item nonresponse is easier to study as inferences about missing information can be made from available OHRQoL information. Findings for item nonresponse may also be partially informative for subject nonresponse based on the assumption that the participants missing all OHRQoL items are similar compared to those missing some items.

The amount of missing information is often reported in studies. For instance, 0.7% of the OHIP item information was found missing, in a clinical study [7] investigating the minimal important difference for the OHIP-49 questionnaire given to prosthodontic patients, described as those who received fixed, removable, partial, or complete tooth replacements [8]. In another study, where the OHIP-49 was administered to a Swedish general population, 2% of the information was missing [9]. Yet, besides the mode of questionnaire administration [10], the previous studies have not focused on factors such as sociodemographic characteristics, and specific items in the questionnaire that might be associated with the level of missing OHRQoL data.

The Dimensions of Oral Health-Related Quality of Life (DOQ) project was an international project, which combined OHRQoL data from 35 individual studies conducted in Croatia, Germany, Hungary, Japan, Slovenia, and Sweden [8]. The DOQ project intended to investigate the number and nature of dimensions that constitute OHRQoL, which is a multidimensional construct [8]. Most OHRQoL instruments, including the OHIP, were based on the Locker's model of oral health [11], which was adapted from the World Health Organization’s (WHO) International Classification of Impairments, Disabilities, and Handicaps model developed in 1980 [12]. However, the 1980 model is no longer valid, as in 2001, the WHO’s International Classification of Functioning, Disability and Health (ICF) became the new international standard to describe disability and health [2, 13]. Originally, the 49 items of OHIP were assigned to seven domains based on the Locker’s model of oral health, however, more recent factor analyses have shown that four highly correlated factors—named Oral Function, Orofacial Pain, Orofacial Appearance, and Psychosocial Impact—can be reliably extracted from the OHIP item pool [14–16]. In fact, John et al. used data from the DOQ project to integrate items from the OHIP-49, the GOHAI, and the OIDP, into the four-dimensional OHRQoL framework [14]. They found the items of the three OHRQoL instruments had overlapping content and could be integrated into the four dimensions.

The DOQ project provided a unique opportunity to study missing OHIP item information from approximately 10,000 people from six countries in different cultural settings [8]. Consequently, the DOQ project provided the sample size and the variety of settings required to investigate missing data for settings typical of OHRQoL assessment. Due to the widespread use of the OHIP-49 [17, 18], we specifically analyzed OHIP-49 data for the current study. Knowledge regarding the relationship of missing information with the demographic factors and the (baseline) level of OHRQoL is essential, as they could be important predictors and confounding variables for association and treatment studies, in which OHRQoL is the main dPRO.

This study aimed to investigate if in the OHIP-49: (i) more missing data occurred when the participants answered more questions, (ii) more missing data occurred in a particular item or set of related items or OHRQoL dimensions namely—Oral Function, Orofacial pain, Orofacial Appearance, and Psychosocial Impact, and if (iii) item missingness was associated with gender, age, and OHRQoL level.

## Methods

### Study design, participants, and OHIP data

We used secondary data from the DOQ project [8]. Out of the 35 studies in the project, 19 of them had some missing OHIP information, and were therefore considered for analyses. A total of 4,847 people participated in the study. Two methods of data collection were employed:

1. Data collection with supervision. The dental patients participating in the studies in Germany, Hungary, Japan, Slovenia, and Sweden had some degree of supervision when they filled in the OHIP-49 questionnaire (18 studies, N = 3,481), and
2. Data collection without supervision. People from the general population in Sweden (N = 1,366) had no supervision, as they received the OHIP-49 questionnaire by mail and completed it at home.

For the purpose of this study, only the 46 OHIP items that were not specifically referring to dentures were used in the analysis. The denture-related items were not relevant for all the participants and these participants may not have answered the denture-related questions; and we could not have differentiated this situation from the missing OHIP information due to the influence of our variables of interest. Additionally, when presenting percentage of missing information, denture-related items presented an obvious problem for the denominator of the calculations.

### Data analysis

To characterize missing data in the two samples with and without supervision, the following descriptive statistics were calculated:

- Proportion of participants with missing OHIP information: number of participants with any missing OHIP items divided by the total number of participants.
- Proportion of missing items in all participants: number of missing items divided by the total number of items among all participants.
- Proportion of missing items in participants with missing OHIP information: number of missing items divided by the total number of items among participants with any missing OHIP items.

We also described distribution of the missing information for subsets of participants based on item location (first/second half of the items), gender, and age (younger/older- according to participants’ median age), and OHRQoL impairment level (less/more—according to median split of the OHIP summary score).

### Aim I: To investigate if more missing data occurred when participants answered more OHIP-49 questions

We fitted a flexible curve (“lowess”, locally weighted scatterplot smoothing) through the 46 proportions of included OHIP items to provide a visual impression (possibly non-linear) of trends of more missing items when more questions are answered. Then, we used logistic regression analysis with missing information as the dependent variable and number of questions completed (OHIP item rank) as the independent variable (ranging from 1 to 46). As the 46 OHIP items were available for each study’s participants, a random-intercept logistic model was used with participants as a random factor. This analysis accounted for multiple missing items occurring within each participant, a situation which could violate statistical independence of the outcome variable. Conceptually, the relationship between the OHIP item rank (indicating the number of questions answered) and missing OHIP item information was analyzed with each study participant having an individual intercept in the regression analysis. OHIP item rank was modelled as a linear variable (1–46).

### Aim II: To investigate if more missing data occurred in a particular item or set of related items (or OHRQoL dimensions)

We used the same random-intercept model again, but instead of OHIP item rank as a linear variable, it was modelled with 45 indicator variables (item 1, *difficulty chewing*, served as the reference category). We also determined the prevalence of missing items for the four OHRQoL dimensions (set of related items) and compared missingness across the four dimensions. Missingness across dimensions was calculated as number of missing items in a particular dimension divided by total number of items in this dimension [result expressed as a percentage]. We also computed the 95% confidence intervals, taking into account that a study participant had several items per dimension by using a bootstrap method with 1000 replications that sampled participants. We also fitted a random-intercept logistic model with the four OHRQoL dimensions as indicator variables, with the base category being “Psychosocial Impact”, the dimension with the largest number of items. Few OHIP items could not be assigned into the four OHRQoL dimensions, these were represented in a fifth miscellaneous category.

### Aim III: To investigate if item missingness was associated with gender, age (younger vs. older), and OHRQoL level

We performed multivariable logistic regression analyses. The logistic regression analyses for the three aims were performed in each of the two samples of participants (with and without supervision). In the participants with supervision, a variable “language” (modelled as indicator variable) was included to adjust for a language-specific effect on the proportion of missingness. For all the logistic regression models, we considered a p-value of 0.001 statistically significant. All analyses were conducted using statistical software package STATA release 14.0 [19].

## Results

### Demographic characteristics

Our sample consisted a total of 4,847 study participants, out of which, 3,481 participants received supervision and 1,366 participants did not receive any supervision while administering the OHIP questionnaire. In Table 1, we summarize the demographic characteristics and the OHIP score of the study participants. Germany contributed the most number of participants, while Slovenia contributed the least. All the study participants who did not receive any supervision were from Sweden. There were also more females than males participating in the studies in both the groups. There were more participants from the general population than from the prosthodontic patient population among those who received supervision. All the study participants without any supervision were from the general population. The mean age of the participants was 49.9 ± 17.11 (range 16–90) years. The summary scores of 46 OHIP items were higher in the participants from the prosthodontic patient populations than the general populations, indicating worse OHRQoL among the dental patients. The summary score among the participants from the general population who did not receive any supervision was higher compared to those from the general population who did.

**Table 1:** Demographic characteristics and OHIP summary score of study participants

Demographic variable	Studies without supervision (N = 1,366)	Studies with supervision (N = 3,481)
*N(%) or Mean (SD)*		
**Country**		
Germany		2,155 (61.9)
Hungary		659 (18.9)
Japan		459 (13.2)
Sweden	1,366 (100)	119 (3.4)
Slovenia		89 (2.6)
Gender*		
Males	611 (44.7)	1,588 (45.6)
Females	744 (54.5)	1,888 (54.2)
**Population type**		
General population	1,366 (100)	2,118 (60.8)
Prosthodontic patients		1,363 (39.2)
**Age**	49.9 (17.11)	49.9 (17.11)
**Summary scores for 46 OHIP item**		
General population		15.5 (20.7)
Prosthodontic patients		36.6 (29.3)
Total summary scores	19.0 (24.5)	23.8 (26.5)

^*^16 Participants had missing information on gender

### OHIP missingness (%) for the overall participants in two studies with and without supervision

**Fig. 1:**
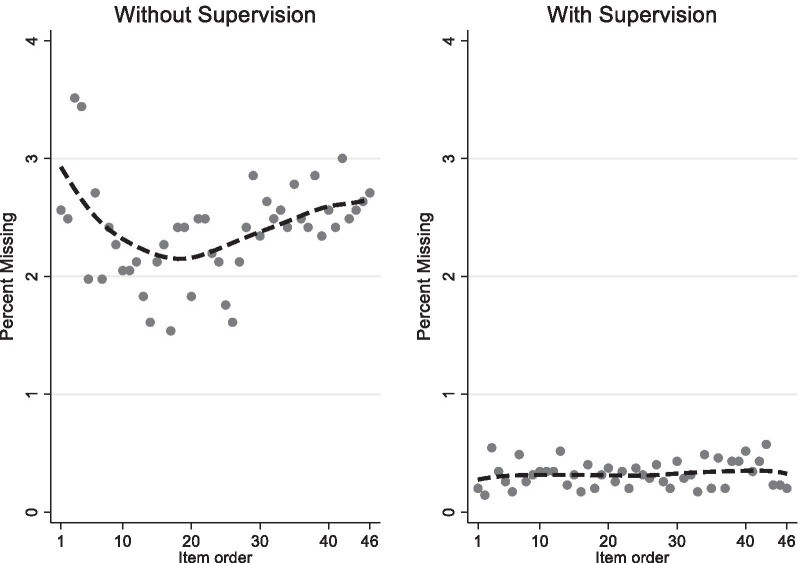
Prevalence of the missing responses for 46 OHIP items in order of how items were filled in by study participants for data collection with and without supervision. Locally weighted scatterplot smoothing (–-) indicates possible trends of missing information with more questions being answered

In studies without supervision, 17.6% of all participants or about every sixth study participant had missing OHIP information. OHIP item missigness was reported 2.4% among all missing items in all participants. It was 13.5% in participants with one or more missing OHIP items. We found that the pattern of missingness was evenly spread across all OHIP items (Fig. 1). The prevalence of missingness for the 46 OHIP items ranged from 1.5 to 3.5% with a median of 2.4 and an interquartile range of 2.1 to 2.6%.

In studies with supervision, 9.3% of all participants or about every tenth study participant had some missing OHIP information. OHIP item missingness was 0.3% among all missing items in all participants. It was 3.5% in participants with one or more missing items. Similar to the studies without supervision, we found the pattern of missingness was evenly spread across all OHIP items, with no specific item having a particularly high or low missingness (Fig. 1). Prevalence of missingness for the 46 items ranged from 0.1 to 0.6% with a median of 0.3 and an interquartile range of 0.2 to 0.4%.

### OHIP missingness (%) for subsets of participants based on item location in the two studies with and without supervision

In the left panel of Table 2, we summarize OHIP missingness (%) in relation to item location in studies without supervision. OHIP item missingness was higher in the first half of the questionnaire (13.4%) compared to the second half of the questionnaire (8.5%) in participants with missing OHIP information. It was slightly higher (2.5%) in the second half of the questionnaire compared with the first half of the questionnaire (2.3%) among all missing items in all participants. OHIP item missingness was also higher (29%) in the second half of the questionnaire compared with its first half (17.1%) among missing items in participants with missing OHIP information.

**Table 2:** Prevalence of OHIP missingness (%) for the overall participants and for subsets of participants based on variables of interest in two studies with and without supervision

	Data collection without supervision			Data collection with supervision		
	Participants with missing OHIP information ^a^	Missing items in all participants ^b^	Missing items in participants with missing OHIP information ^c^	Participants with missing OHIP information ^a^	Missing items in all participants ^b^	Missing items in participants with missing OHIP information ^c^
**Variables of interest**						
*Item location*						
First half of the questionnaire	13.4	2.3	17.1	5.2	0.3	5.9
Second half of the questionnaire	8.5	2.5	29.0	5.4	0.3	6.3
*Age*^*†*^						
Younger	11.3	1.0	8.7	6.8	0.2	3.1
Older	23.1	3.7	16.2	11.9	0.4	3.4
*Gender*						
Male	14.2	1.4	9.8	8.8	0.3	3.4
Female	19.9	3.2	15.8	9.7	0.3	3.3
*OHRQoL impairment*^*†*^						
Less	14.9	2.6	17.6	5.6	0.2	3.7
More	20.4	2.1	10.4	13.2	0.5	3.4

Refer to the definition listed under data analysis (a) – (c) for column labels:

^a^Number of participants with any missing OHIP items divided by the total number of participants

^b^Number of missing items divided by the total number of items among all participants

^c^Number of missing items divided by the total number of items among participants with any missing OHIP items

^†^Median split is applied

The left panel of Fig. 1 displays a fitting curve showing a U-shaped relationship between increasing OHIP information collected and the prevalence of missing information. We found that OHIP item missingness was larger at the beginning and the end of the survey compared to the middle of the survey. Altogether, these differences were minimal (± 1%) compared to the overall value (2.4%). We did not find a notable linear trend between more questions answered and missing data.

Table 3 shows the findings for a regression model with one order as (one) linear variable and a second regression model with all 46 items modelled as indicators variable. Because we have 45 variables in this model, a median, IQR etc. is shown for the size of the 45 odds ratios. The odds ratios (ORs) from the logistic regression analysis were interpreted as risk ratios (RRs) because missing OHIP items were rare. For example, an OR (or RR) of 1.05 would indicate that the risk of missing information increases by 5% when an additional item is asked in the questionnaire. A p-value of 0.001 or less was considered statistically significant. The left panel of Table 3 shows that a 10-unit item increase in item order (e.g., from item #3 to #13) was associated with a 5% risk increase (OR, 95% CI: 1.05, 0.99–1.10). Overall, we found a weak and statistically non-significant association between item order and OHIP item missingness in studies without supervision.

**Table 3:** Four random-intercept logistic regression models relating order of 46 OHIP items as a linear variable or an indicator variable to missing OHIP item responses

		Data collection without supervision	Data collection with supervision
		General population participants in Sweden	Patients and general population participants in Germany, Hungary, Japan, Slovenia, Sweden
	Odds ratio (95% confidence interval)		
Random effects model with item order as linear variable	Rank order	1.05 (0.99–1.10)	1.04 (0.97–1.11)
	Intercept	0.01 (0.00–0.00)	0.00 (0.00–0.01)
Random effects model with item order as indicator variable	Rank order summary^‡^		
	Median	0.89	1.61
	IQR^*^	0.69–1.001	1.15–2.08
	Range	0.37–1.92	0.70–3.06
	Intercept	0.00 (0.00–0.00)	0.00 (0.00–0.00)

^*^Interquartile range, ^‡^Summary of 45 estimated ORs of indicator variables (confidence intervals not shown)

In the right panel of Table 2, we summarize OHIP missingness (%) in relation to item location in studies with supervision. OHIP item missingness was slightly higher in the second half of the questionnaire (5.4%) compared to the first half of the questionnaire (5.2%) in participants with missing OHIP information. 0.3% missingness was reported in both parts of the questionnaire among all missing items in all participants. OHIP item missingness was higher (6.3%) in the second half of the questionnaire compared with its first half (5.9%) among missing items in participants with missing OHIP information. Overall, in the studies with supervision, differences in OHIP missingness between the first and the second half of the questionnaire in all the subgroups were lower compared to studies without supervision.

The right panel of Fig. 1 displays a slightly curved relationship between more OHIP information gathered, and the prevalence of missing information. We did not find a notable linear trend, indicating that OHIP item missingness did not increase when the participants answered more items.

The right panel of Table 3 shows that a 10-unit item increase in item order (e.g. from item #3 to #13) was associated with a 4% risk increase (OR, 95% CI: 1.04, 0.97–1.11). Similar to the other group, we found a weak and statistically non-significant association between item order and OHIP missingness in studies with supervision. Adjustment for the “language” did not change the results. When we considered the participants with and without supervision together, we found a low likelihood of more OHIP item missingness with more questionnaire items answered.

### OHIP missingness (%) for subsets of participants based on items or set of items or OHRQoL dimensions (Oral Function, Orofacial pain, Orofacial Appearance, and Psychosocial Impact) in the two studies with and without supervision

The left panel of Fig. 1, shows the prevalence of missing items in studies without supervision ranged between 1.5 upto 3.5%. In the lower left panel in Table 3, we summarized findings from random-intercept logistic regression models using the 46 OHIP items as indicator variables. When we examined 45 indicator variables for 46 OHIP items (the reference category was item #1), the risk of missing information increased by 91% or decreased by 63% when it was extreme. We found a weak and statistically non-significant association between OHIP item missingness and any particular OHIP item.

In the left panel of Table 4, we summarized the prevalence of missing OHIP information based on the four OHRQoL dimensions in studies without supervision. The prevalence estimates for this group ranged between 2.0 and 2.6% within the four dimensions.

**Table 4:** Prevalence of missing dimensional OHIP information corresponding to the four OHRQoL dimensions

OHRQoL dimensions	Data collection without supervision	Data collection with supervision
	Participants from the general population in Sweden	Participants from the patient population and the general population in Germany, Hungary, Japan, Slovenia, Sweden
	Prevalence in % (95% confidence interval)	
Oral function	2.3 (1.7–2.9)	0.3 (0.2–0.3)
Orofacial pain	2.0 (1.4–2.7)	0.3 (0.2–0.4)
Orofacial appearance	2.5 (1.9–3.1)	0.4 (0.3–0.4)
Psychosocial impact	2.6 (1.8–3.3)	0.3 (0.3–0.4)
Symptoms	2.1 (1.5–2.7)	0.3(0.2–0.4)

We used the four OHRQoL dimensions as indicator variables with the dimension of “psychosocial impact” as the reference dimension in our regression models. The OR estimates were below or close to 1.0 for each OHRQoL dimension.

The right panel of Fig. 1 shows the prevalence of missing items in studies with supervision ranged between 0.1 and 0.6%. When we examined 45 indicator variables for 46 OHIP items (the reference category was item #1), the risk of missing information increased by 206% or decreased by 30% when it was extreme. Similar to the studies without supervision we found weak and statistically non-significant association between OHIP item missingness and any particular OHIP item.

In the right panel of Table 4, we summarized the prevalence of missing OHIP information based on the four OHRQoL dimensions in studies with supervision. The prevalence estimates for this group ranged between 0.3 and 0.4% within the four dimensions.

Our logistic regression analysis resulted in OR estimates ranging from 0.75 to 1.03 for each OHRQoL dimension. None of the ORs were statistically significant. Overall, when we compared the studies with and without supervision, we found no consistent pattern of missingness for individual OHIP items or for the four OHRQoL dimensions. No specific item or OHRQoL dimensions had a significant association with OHIP missingness.

### OHIP missingness (%) for for subsets of participants based on gender, age, and OHRQoL level in the two studies with and without supervision

In the left panel of Table 2, we summarized OHIP item missigness based on age, gender, and OHRQoL impairment level in studies without supervision. OHIP item missingness was greater in older and female participants in all the subsets of participants. OHIP item missingness was greater in those with more OHRQoL impairment among the participants with missing OHIP information, While, in the other two subsets, missingness was more among those with less impairment. In other words, we did not find a consistent pattern of OHIP item missingness depending upon the levels of OHRQoL impairment.

In the left panel of Table 5, we presented findings from logistic regression analysis based on demographic variables and OHRQoL impairment level in studies without supervision.Older persons and females were 5.47 and 2.66 times more likely to have missing items than younger and male population, respectively. Worse OHRQoL was not statistically significant and there were no differences of missingness according to the OHRQoL impairment (OR, 95% CI: 1.34, 0.82–2.18).

**Table 5:** Two random-intercept logistic regression models showing the associations between OHIP item missingness and age, gender, and OHRQoL levels

Predictor variables	Data collection without supervision	Data collection with supervision
	Participants from the general population in Sweden	Participants from the general population and the patient population in Germany, Hungary, Japan, Slovenia, Sweden
	Odds ratio (95% confidence interval)	
Older age	5.47 (3.19–9.39)	1.70 (1.29–2.24)
Female gender	2.66 (1.59–4.45)	1.09 (0.84–1.42)
More OHRQoL impairment	1.34 (0.82–2.18)	2.65 (2.00–3.52)
Intercept	0.00 (0.00–0.00)	0.00 (0.00–0.00)

In the right panel of Table 2, we summarized OHIP item missigness based on age, gender, and OHRQoL impairment level in studies with supervision. We found relatively less OHIP item missingness within the two age and gender groups compared to the studies without supervision. OHIP item missingness was still more in older and female participants in most subsets of participants, but the differences in proportions between male versus female and younger versus older participants were relatively less than those without any supervision. Similar to the studies without supervision, we did not find a consistent pattern of OHIP item missingness across the levels of OHRQoL impairment. We found more OHIP item missingness in those with more OHRQoL impairment in two subsets of participants, and in one subset the missingness was more in those with less impairment.

In the right panel of Table 5, we presented findings from logistic regression analysis based on demographic variables and OHRQoL impairment level in studies with supervision. Older persons and participants with more OHRQol impairment were 1.70 and 2.65 times more likely to have missing items than their counterparts, respectively. Gender was not statistically significant and there were no differences of missingness by gender (OR, 95% CI 1.09, 0.84–1.42).

## Discussion

Study participants from general and dental patient populations in six countries did not find the OHIP-49 questionnaire burdensome. OHIP item missingness did not depend on a particular OHIP item or set of related items (or OHRQoL dimensions). It also did not depend on if the participants responded to a greater number of OHIP items. We did not find a consistent pattern of the influence of sociodemographic factors and OHRQoL impairment level on OHIP missingness. Overall, the amount of missing OHIP information was low making any potential influence likely small in magnitude.

### Comparison with literature

The OHIP-49 has been widely used and adapted to several cultures. Our findings of low burden and high acceptability of OHIP has been demonstrated in previous studies [17, 20–22]. For example, very low subject and item nonresponse was found for the German-version of OHIP (OHIP-G) [21]. In 98% of the participants, the OHIP-G49 and dimension scores could still be calculated. We also found low OHIP item missingness for all participants and for most subsets of participants based on variables of interest in the studies with and without supervision.

Based on the four-dimensional OHRQoL model, we also investigated missingness across the dimensions of Oral Function, Orofacial pain, Orofacial Appearance, and Psychosocial Impact. Originally, the 49 OHIP questions were grouped into seven domains, and the seven domain scores characterized the broader impact from oral diseases [11, 17]. However, recent studies suggest that the OHIP items can be grouped into four correlated dimensions, and OHRQoL assessment results in a score for the overall impact and four scores for dimensions [11, 14–16]. Our results were unexpected considering that our study sample majorly consisted of prosthodontic patients because usually for an oral condition, one or more areas of impact (or dimensions) are more prominent than the other areas. Hence, the dimension scores vary across different oral conditions. Typically, in prosthodontic patients functional impact predominates over the other areas of impact [23]. While, in patients with periodontitis, functional, pain-related, and psychosocial impacts predominate [10, 24–26]. In this study, OHIP item missingness was not high for any particular OHRQoL dimension. In fact, the pattern of missingness was evenly spread out across all dimensions.

We did not formally compare missingness in the participants based on the level of supervision, yet overall missingness for the participants in studies with and without supervision was low. Reissmann and others also found a small amount of missing OHIP information when comparing different methods of administration (personal interview, telephone interview, self-administered questionnaire) among German prosthodontic patients [26]. They found that the method of administration did not substantially influence OHIP scores in prosthodontic patients. Contrarily, Desai et al. [10], found that there was a significant difference in OHIP summary scores based on the method of administration. They observed higher summary scores (poorer OHRQoL) in self-administered questionnaire compared to telephone administration among British patients with chronic periodontitis. Higher scores (more impairment) have also been reported when health-related quality of life (HRQoL) questionnaire was self-administered or mailed to the participants compared with interviewer administered questionnaire [27–30]. In fact, more missing information was observed when the 36-Item Short Form Health Survey (SF-36) was administered through mail compared to interview-administration [29].

The study findings indicated OHIP item missingness was greater in older and female participants in all the subsets of the participants. However, we did not find a consistent pattern of OHIP item missingness across all subsets in relation to high or low OHRQoL impairment levels. Our logistic regression analysis findings suggested differences in missingness between male and female participants and older and younger participants, and no differences in missingness based on OHRQoL impairment levels in studies without supervision. On the other hand, the findings suggested differences in missigness based on age and OHRQoL impairment levels, and no differences based on gender in studies with supervision. The missingness did not hinder us from characterizing OHRQoL for women, older people, or those with more OHRQoL impairment. Because the base rate of missing items is low, likely all multipliers, i.e., odds ratios, did not result in a substantial number of missing items. For example, the prevalence of missing items in the younger participants with supervision is 0.2% (of all items) and even if the prevalence doubles (odds ratio of ~ 2) to 0.4% there is still 99.6% of the item information available. For the unsupervised Swedish participants, these numbers are 1% and 3.7%, respectively. From a relative point of view, this is a substantial increase; however, from an absolute point of view, there is still more than enough OHRQoL information to characterize the participants’ impairment. We investigated item-level missingness, but we can compare our findings with other studies that recorded participant-level missingness. Turrell and others [31] examined the contribution of neighbourhood disadvantage and socioeconomic characteristics to OHRQoL. They achieved a moderate individual-level response rate of 69.4% that was inversely related to the levels of neighbourhood disadvantages. Other studies also suggest that socioeconomic factors are associated with nonresponse to OHRQoL questionnaires [31–33].

### Strengths and limitations of the study

Documenting missing data is common for studies investigating OHRQoL using the OHIP-49 questionnaire. Previous studies have looked at the influence of the factors such as change in the order of OHIP items [34] and the modes of administration on overall OHIP scores [10]. Our study however is distinct from these studies as it is the first study to specifically investigate missingness in the OHIP questionnaire based on multiple variables of interest. Our study also had a large sample size, with data from 35 individual studies from six different countries, thus representing relevant clinical and general populations from different cultures. Additionally, previous researchers have shown strong correlation and overlap in item content among the three commonly used OHRQoL instruments – GOHAI, OHIP, and OIDP [14, 35–39]. This evidence thus suggests that findings from the current study would be applicable to OHRQoL instruments other than the OHIP as they measure the same construct. We recommend further investigations of missingness in other OHRQoL instruments such as GOHAI and OIDP. Our study methods can be applied to explore missingness based on several variables of interest in other OHRQoL instruments. The findings would help clinicians and researchers assess if these instruments are burdensome for respondents and if they can be improved.

This study has some limitations. Potential biases such as social desirability bias and selection bias may occur with OHRQoL data, however, we do not expect them to influence missingness in the OHIP questionnaire. We studied supervised and unsupervised participants because the method of administration can influence the number of missing OHIP items [10]. We investigated our variables of interest within each of the groups however our study did not involve a formal comparison between the supervised and unsupervised participants. Both the groups differed in sociodemographic characteristics. Within each group, the study participants can be different compared to non-responders considering many variables (selection bias), but a noteworthy influence of selection bias in terms of missing OHIP information is unlikely. As we demonstrated in this study, the amount of missingness is generally small. Overall, we did not find a consistent pattern in the influence of sociodemographic and OHRQoL impairment level scores. Also of note is that we examined OHIP item missingness based on some important demographic variables, however factors such as socioeconomic status and race/ethnicity were not included in our current analysis. These variables might be of interest to dental providers and researchers, and we recommend studies in the future to examine the influence of these factors on OHIP item missingness. Lastly, our analysis includes data from multiple studies conducted in six different countries and country-specific random effects occurrence can potentially occur. However, as mentioned before, the amount of missingness is small and there is no consistent pattern across the groups, thus influence of the random effects on the study results is unlikely.

## Recommendations and clinical relevance

As the OHIP-49 questionnaire is the most widely used dental patient-reported outcome measure (dPROM) our findings are important for dental professionals [4] who seek to use the questionnaire, but they may hesitate due to more burden on the respondents. Our findings showed a small amount of missingness in general, without any consistent pattern of influence of gender, age, and OHRQoL level on OHIP missingness. Overall, missingness was not more for any particular item or set of related items (or OHRQoL dimensions). Low OHIP item missingness in a large study sample with representation from different populations is promising for further use of the OHIP-49 questionnaire in general and dental patient populations. Although shorter versions of OHIP are available (OHIP-5 and OHIP-14) and reduce time-, effort-, and resource- related burden on respondents and administrators [17]; the longer version measures OHRQoL with greater precision. When missing data occur, we recommend referring to the best practices with handling and reporting missing outcome data in patient-reported outcome measures (PROMs) [40]. For example, when researchers encounter a small amount of missing data, it can be imputed using a regression method [41]. In the future, the findings and methodologies from this study may be extended to other research studies to investigate OHIP item missingness in other OHRQoL questionnaires and dPROMs in general.

## Conclusions

We found low missingness across individual items or sets of related items in the OHIP-49 questionnaire; and it was not more likely for more missing information when the participants answered more questions. Also, we did not observe a consistent pattern in the influence of sociodemographic and OHRQoL magnitude on missing OHIP information. Overall, we did not find the length of OHIP-49 to be burdensome for further application in general and dental patient populations.

## Data Availability

The datasets used and analyzed during the current study are available from the corresponding author upon reasonable request.
